# Caries Risk Assessment and Management in Europe: The Multi-Country Observational CARMEN Study

**DOI:** 10.3390/dj14020126

**Published:** 2026-02-23

**Authors:** José Frias-Bulhosa, Agnieszka Mielczarek, Nikolai Sharkov, Maria Gaveli, Ana Luísa Costa, Alberto Ogalla, Pierre-Marie Voisin, Sylvain Levet, Jean-Noel Vergnes

**Affiliations:** 1 Health Sciences Faculty, Fernando Pessoa University, 4200-150 Porto, Portugal; jfrias@ufp.edu.pt; 2 Department of Conservative Dentistry, Medical University of Warsaw, 02-091 Warsaw, Poland; agnieszka.mielczarek@wum.edu.pl; 3 Department of Paediatric Dental Medicine, Faculty of Dental Medicine, Medical University, 1431 Sofia, Bulgaria; nikolai@sharkov.eu; 4 Palaio Faliro, 17561 Athens, Greece; maria_gaveli@hotmail.com; 5 Faculty of Medicine, University of Coimbra, 3004-531 Coimbra, Portugal; acosta@fmed.uc.pt; 6 Pierre Fabre, 81100 Castres, France; alberto.ogalla@gmail.com (A.O.); pmvoisincd@gmail.com (P.-M.V.); sylvain.levet@pierre-fabre.com (S.L.); 7 Toulouse Dental Faculty, Toulouse University, 31013 Toulouse, France; 8 Toulouse University Hospital (CHU de Toulouse), 31300 Toulouse, France; 9 INSERM CERPOP, 31000 Toulouse, France; 10 Faculty of Dental Medicine and Oral Health Sciences, McGill University, Montreal, QC H3A 0G4, Canada

**Keywords:** caries risk management, dental caries, dental care, dentists, Europe

## Abstract

**Background:** This observational ambispective longitudinal international study explored dentists’ practices and patient records concerning dental caries risk management across four European countries (Bulgaria, Greece, Poland and Portugal). **Methods:** Dentist volunteers recruited patients needing caries risk management, either through preventive or curative measures, from their regular practice. Analyses focused on assessing dentists’ practices in caries risk assessment and management, along with gathering information on patient and dentist characteristics, oral health assessments, and caries risk evaluation. **Results:** A total of 51 dentists recruited 1008 patients. Across the countries studied, caries risk assessment and management methods varied, with fewer than 15% of dentists using standardized tools. Primary assessment methods included oral examinations and medical interviews, while nutritional and fluoride intake assessments were less common, and salivary or microbiological tests were rare. There was an inverse association between the risk of dental caries and patients’ socioeconomic status. Specific university training on caries risk showed a positive correlation with adherence to recommendations. **Conclusions:** Our findings emphasize the importance of clinicians adapting their approaches to individual patient needs in caries risk assessment and management. However, the wide array of available risk assessment tools presents a challenge, underscoring the necessity of integrating biopsychosocial models into dental practice to effectively deliver personalized care.

## 1. Introduction

Dental caries is a biofilm-mediated, sugar-driven, multifactorial, dynamic disease that results in the phasic demineralization and remineralization of dental hard tissues [1]. Frequent consumption of fermentable carbohydrates promotes the growth of bacteria within the dental biofilm. Understanding the interplay of these two factors underscores the importance of promoting oral health through supportive practices and dietary choices. Additionally, various biological factors including salivary dysfunction, as well as environmental determinants such as socioeconomic status and limited access to dental care, can exacerbate caries risk [2,3]. Managing dental caries in individuals with multiple risk factors presents a formidable challenge, necessitating a delicate equilibrium between treatment and prevention strategies [4,5].

The management of dental caries entails a comprehensive approach aimed at controlling both protective factors and mitigating pathological risk factors associated with caries development [6]. Contemporary dental practice underscores the importance of personalized care and tailored advice, grounded in a thorough assessment of caries risk. This assessment serves as a pivotal tool in not only gauging susceptibility to carious lesions but also devising a bespoke strategy to foster preventive behaviors while optimizing the scheduling of dental check-ups. Increasingly, Caries Risk Assessment (CRA) is recognized as the cornerstone of effective caries management across all age groups [7,8]. There are several models for assessing caries risk, which incorporate between 9 and 14 socio-demographic, behavioral, clinical, salivary, and/or microbiological factors. These include, but are not limited to, the National University of Singapore Caries Risk Assessment (NusCra), Caries Management by Risk Assessment (CAMBRA), Caries Risk Assessment by the American Dental Association, the Caries Assessment Tool by the American Academy of Pediatric Dentistry, Cariogram, and PreViser [9].

By systematically evaluating individual risk profiles, clinicians can offer targeted interventions tailored to each patient’s unique needs, thereby enhancing the efficacy of preventive measures and optimizing oral health outcomes.

Minimal Intervention Dentistry (MID) emphasizes the importance of individualized care and patient involvement, with CRA serving as a cornerstone in preventive and therapeutic dental approaches. Since the early 2000s, various models of CRA have been developed to aid clinicians in identifying individuals predisposed to future dental caries. However, despite their widespread use in dental literature, a systematic review in 2018 underscored the limited predictive accuracy of standardized CRA models in reliably predicting caries lesions [9]. Nevertheless, the adoption of CRA by dentists reflects a commitment to practicing minimally invasive dentistry, prioritizing patient well-being and preserving dental health.

Despite significant epidemiological advancements in the reduction in dental caries over the past three decades, it remains a prevalent public health concern [10]. While guidelines have been established for the early identification of high-risk caries patients and the implementation of preventive strategies for at-risk populations [4], there exists a paucity of data regarding the clinical practices of dental professionals in Europe.

In this context, the CARMEN study, which stands for CAries Risk assessment and Management for European Networks, aims to investigate the prevalence of CRA practices among European dentists. Additionally, we seek to explore the methodologies employed by dentists in assessing caries risk, particularly in instances where reliance on software programs or standardized models from dental associations is not feasible. By examining the relationship between CRA approaches and patient data, our study aims to shed light on the nuanced decision-making processes employed by dental professionals in delivering patient-centered care. The goal is to translate clinical insights into relevant, locally impactful programs that reinforce prevention-focused care.

## 2. Materials and Methods

### 2.1. Study Design and Ethics

This was an international observational ambispective longitudinal study, with collection of patient data conducted with dental clinicians in four European countries: Bulgaria, Greece, Poland and Portugal. Data were collected from 2019 to 2021 and facilitated by the partnership with Pierre Fabre Laboratory. However, the latter part of data collection coincided with the onset of the COVID-19 pandemic.

Each participating country was assigned one main investigator responsible for overseeing activities within their respective country. Prior to study commencement, a comprehensive training session was conducted to ensure consistent understanding of research protocols. Additionally, a Contract Research Organization (CRO) was engaged to ensure adherence to good clinical practices and data quality.

To safeguard data confidentiality, a secure centralized online platform compliant with the General Data Protection Regulation was established for data collection. All participants were informed of the study protocol, and written consent was obtained. Furthermore, ethical considerations were addressed at each local level through the CRO, thereby ensuring compliance with ethical standards and protection of participant rights throughout the study. Portugal and Poland were the only two countries that needed ethical consideration and approval (number AKBE/266/2019 (24 June 2019) for Poland and 031-CE-2020 (17 February 2020) for Portugal).

### 2.2. Study Population

Dentists were selected using a random sampling process based on national source files specific to each country. This approach constitutes a form of cluster sampling, where each country was considered a cluster, and dentists were randomly selected within these clusters. Participating dentists were required to include patients from their regular practice. After assessment of the investigators’ workload, particularly regarding patient follow-up, an average of 20 patients per dentist was established. Assuming that approximately 15 dentists per country would be actively including patients, the study aimed to enroll ~300 patients per country, which was expected to provide an acceptable accuracy of ~6–7% (based on a binomial confidence interval approach). To ensure this number could be met and bearing in mind that around 70% of dentists would be active during the inclusion period, approximately 20 were to be recruited per country. Consecutive patients, adults or children with at least one permanent tooth identified by the dentist as needing caries risk management, either through preventive or curative measures, were eligible for inclusion. Exclusion criteria included children without at least one permanent tooth (consistency with the caries risk), patients who did not consent to participate, and patients already enrolled in another clinical study protocol.

### 2.3. Data Collection

Data were collected through clinical records and patients visits with an online centralized platform specifically designed for this purpose. The platform facilitated data entry, dentist management, and study monitoring. Questionnaires were completed either by the dentist or their assistant. Guidelines for data validation and addressing missing or inconsistent data were outlined in the data handling manual, which was validated by the steering committee. Data review and quality assurance were made at the end of each data collection period by a data manager, who controlled data consistency and sent queries to the dentists if it was necessary.

Information regarding the characteristics of participating dentists and patient volunteers was collected. These details included patient characteristics, oral health assessments, caries management, and patient education at the time of enrollment. Additionally, retrospective data on previous dental care provided to patients over the past three years were gathered at enrollment, while prospective data on dental care provided one-year post enrollment were obtained from patient records.

### 2.4. Statistical Analyses

Descriptive analyses for qualitative variables encompassed various aspects, including sample size, frequency, and percentage of each category, as well as the number of missing values. These analyses were particularly focused on variables pertinent to CRA, such as the percentage of dentists adhering to specific recommendations, the distribution of recommendations followed, and the percentage of dentists implementing CRA, preventive measures, counseling, and early management of caries lesions.

Quantitative variables were described using cross-tabulations, providing insights into various patient subgroups (e.g., age categories) and the entire study population. This included information on sample size, range, and mean with SD, along with the number of missing data points.

Association of active caries lesions and CRA and socioeconomic status for patients of all countries was assessed with Chi-square tests. The SAS software program (version 9.4, SAS Institute, Cary, NC, USA) was employed for data management and statistical analysis. A significance level of 0.05 (α = 0.05) was set for all tests conducted.

Missing data were detailed for each variable in a descriptive table.

## 3. Results

### 3.1. Dentists’ Characteristics and Practices

**Figure 1:**
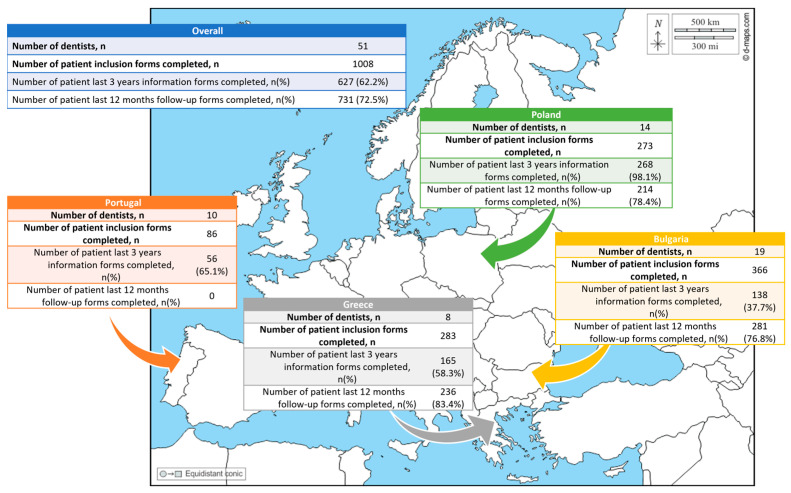
Geographical repartition of dentists and patients.

Fifty-one dental clinicians agreed to take part in the study (19 from Bulgaria, 8 from Greece, 14 from Poland and 10 from Portugal) (Figure 1). Out of the total, 43 dentists recruited at least one patient (84%), 18 in Bulgaria (95%), 8 in Greece (100%), 14 in Poland (100%) and 3 in Portugal (30%). Most of them (63%) were female (Table 1).

The mean age of dental professionals was 44 years (±12), with an average duration of practice of 19 years. The mean age of dentists was consistent across countries, ranging from 40.7 (±5.9) in Portugal to 47.1 (±10.4) in Greece. Urban practice was predominant, with 62% of dentists practicing in urban areas, although this proportion was lower in Portugal. Most dentists held specializations in pediatric dentistry (15.7%), dental surgery (15.7%), and conservative stomatology/and endodontics (15.7%). Notably, 48% had received specific education on caries risk management at university, with varying rates observed between countries: 78.6% in Poland, 60% in Portugal, 28.6% in Greece, and 26.3% in Bulgaria. Nearly all dentists reported engaging in continuing dental education, although only 72% considered themselves up-to-date, with higher proportions in Greece (85.7%) and lower proportions in Bulgaria (68.4%) (Table 1).

In all countries, the majority of dentists reported relying on recommendations for caries risk management (more than 55.6%) and 57.1% claimed to conduct caries risk assessments at every patient’s dental visit (Table 2).

Dentists reported conducting patient risk assessments for dental caries, typically preferring an annual frequency (53.1%), except in Greece, where assessments were reportedly performed at each visit. Across all dentists, more than half reported routinely conducting systematic CRA (Table 2). However, variations were evident between countries, with 100% of participants in Greece, 77.8% in Portugal, 47.4% in Bulgaria, and 35.7% in Poland reporting this practice (Table 2). The CRAs primarily relied on oral examination (98%), medical interview (79.6%), nutritional assessment (71.4%), radiographic examination (67.3%), topical fluoridation (51%), and fluoride intake (40.8%). Notably, the utilization of fluoridation and fluoride intake was more widespread in Poland compared to other countries (85.7% and 71.4%, respectively, vs. less than 60% in other countries). Salivary tests and the use of CRA scales and software were infrequent (Table 2).

**Figure 2:**
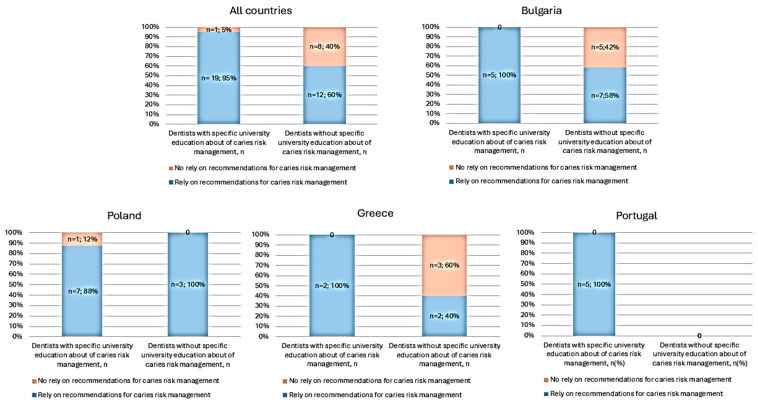
Association between specific university training on caries and adherence of dentists to recommendations in all countries and per each country.

There was a significant association between having received specific education on caries risk management at university and adherence to guidelines in routine practice (*p* < 0.001). Ninety-five percent of those who had received education on caries risk management at university adhered to caries risk management recommendations, compared to 60% of those who had not received such education (Figure 2).

This trend was particularly high in Portugal, Greece and Bulgaria, with an association of 100% between specific education and the reliance on recommendations. Only Poland had a dentist with specific education who does not rely on recommendations. However, the proportion of dentists without specific university training was higher in Greece and Bulgaria, and among these dentists, 40% and 58% (in Greece and Bulgaria, respectively) relied on recommendations (Figure 2).

### 3.2. Patients’ Characteristics

The total patient sample comprised 1008 individuals, with a mean age of 35.2 years (±19.7). There were 366 from Bulgaria, 283 from Greece, 273 from Poland, and 86 from Portugal (Figure 1). Among these patients, the majority (56%) were female (Table 3). Portugal started data collection later, and during 2020, the dental offices were suspended due to the COVID-19 pandemic. This limited inclusion and the collection of prospective follow-up data.

Nearly 80% of patients had recent caries experiences, with rates varying across countries: 69% in Bulgaria, 75% in Greece, 90.7% in Portugal, and 96% in Poland. Despite this, over 90% of all patients reported daily teeth brushing, with 98.5% of Polish patients and 93% of Portuguese patients adhering to this practice. Additionally, 82.7% of patients claimed to use fluoride toothpaste. At the inclusion visit, 53.3% of patients exhibited visible plaque on their teeth, while 44.2% had active carious lesions, and 38.4% presented with deep pits and fissures (Table 3). Direct restoration was the most common treatment provided during the inclusion visit, administered to almost half of the patients.

**Figure 3:**
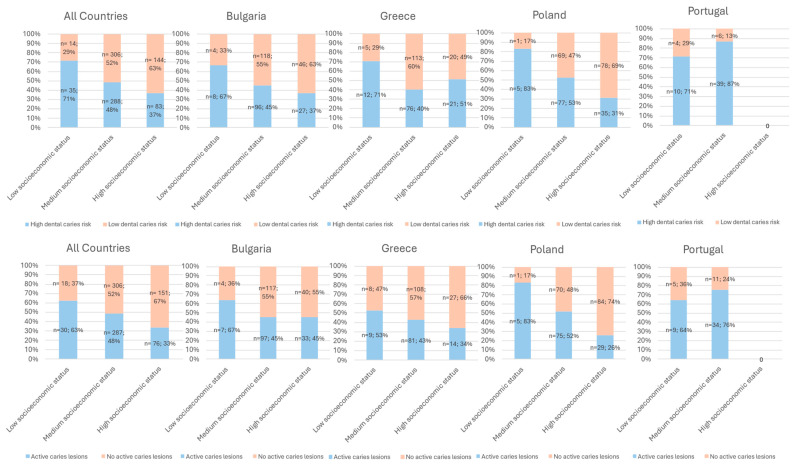
Association between dental caries risk and active caries lesions and socioeconomic level for patients across all countries and by country.

The risk of dental caries and the presence of active carious lesions both appeared to exhibit an inverse association with socioeconomic status (Figure 3).

Active carious lesions were observed in 33%, 48%, and 63% of patients classified as having high, medium, and low socioeconomic status, respectively. Correspondingly, dentists reported a high caries risk in 37%, 49%, and 71% of patients within these socioeconomic strata (Figure 3). Despite these findings, few disparities were evident across countries. Notably, in the Portuguese population, individuals with low socioeconomic status exhibited lower rates of active carious lesions and dental caries risk compared to those with medium socioeconomic status. Conversely, in Greece, individuals with high socioeconomic status displayed a higher correlation with increased dental caries risk compared to those with medium socioeconomic status.

During the dental visits, most patients received advice on oral hygiene or diet, though there was variation among countries: 98.9% in Bulgaria, 92.2% in Greece, 78.3% in Poland, and 64% in Portugal. Among Bulgarian dentists, 68.3% reported that more than half of their patients exhibited a positive attitude towards their care and a willingness to cooperate. This proportion was 59.1% for dentists in Poland, 46.3% in Greece, and 29.1% in Portugal (Supplementary Tables S1 and S2).

A high level of oral health was more prevalent during the year following inclusion compared to the three years prior (see Table 4 and Table 5). Overall, the proportion of patients with a high level of oral health ranged from 28% during the preceding three years to 32% during the follow-up period. This increase was consistent across all countries: in Bulgaria, 29% of patients exhibited a high level of oral health three years before inclusion, compared to 35% one year after; in Greece, the percentages were 8.5% versus 18%; and in Poland, 42% versus 47%.

Differences emerged in the patterns of caries care provided between countries. Fluoride application was more common in Greece and Poland than in Bulgaria (54% and 67% and 3% during the three years before inclusion and 47%, 48% and 5% during the follow-up, respectively). Remineralization procedures were more frequently performed in Poland compared to other countries (20% vs. less than 4.3% three years before and 21% vs. less than 16% one year after inclusion). However, the frequency of remineralization increased in Greece from 1.2% three years before inclusion to 16% one year after.

There were no changes in patient education provided by dentists between three years before and one year after inclusion. Bulgaria provided more counseling than Greece and Poland (95% vs. 85% and 66% during the three years before inclusion and 85% vs. 69% and 57% one year after inclusion, respectively). Assessment of the patient’s attitude toward care and their ability and willingness to cooperate was higher in Bulgaria compared to elsewhere (Table 4 and Table 5).

## 4. Discussion

Across the four European nations under scrutiny, notable diversity is evident in the approaches taken by dental clinicians towards caries risk management and counseling. However, dentists who had received specific university training on caries risks demonstrated heightened adherence to caries risk recommendations across all participating countries.

Not surprisingly, our findings underscore a discernible association between patients’ socioeconomic status and the level of dental caries risk, as well as the presence of active carious lesions. Specifically, individuals from lower socioeconomic backgrounds exhibited a higher likelihood of encountering these oral health issues. Moreover, our study revealed a noteworthy trend indicating a higher frequency of overall assessments of oral dental health one year after patient inclusion compared to assessments conducted three years prior. This temporal variation might suggest potential progression towards more vigilant and proactive approaches to oral health evaluation and management among dental practitioners. It is also possible that this effect is directly linked to the participation of dentists who are more attuned to these issues, encouraging their involvement in this study.

### 4.1. Dentists’ Specificities and Practices About Caries Risk Assessment

As expected, an overall positive association was observed between specific university training in caries and adherence to recommendations. However, despite some dentists without specific university training in caries risk professing to rely on recommendations, a gap was apparent in Bulgaria and Greece, where a high proportion of dentists lacking caries-focused university education did not rely on caries risk recommendations. While these findings highlight the potential importance of university education in shaping evidence-based practices, the observational design of our study limits causal inference. This association should be interpreted with caution. It is also worth noting that although 94% of dentists overall pursue continuing education, only 72% consider themselves up-to-date, suggesting a need for more targeted opportunities in CRA.

Dentists claimed to primarily base their practices on guidelines from Learned Societies, such as the European Academy of Pediatric Dentistry (EAPD) [11], American Academy of Pediatric Dentistry (AAPD) [12], and American Dental Association (ADA) [13]. In some cases, recommendations specific to caries prevention and treatment, such as CAMBRA [14] and ORCA [15], are consulted, while techniques such as dental glass sealant are marginally considered. Local recommendations, especially in Poland, are also followed.

The process of caries risk assessment varied depending on the country. While an oral exam was systematic across all countries, a medical interview was less commonly performed in Portugal, possibly due to the specific nature of this dentist cohort. Additionally, there were disparities in the use of radiographic examination, which was less frequent in Bulgaria, likely because this service was not covered or was predominantly paid for by the patient [16]. Polish dentists showed greater sensitivity to fluoridation and fluoride intake compared to other countries, possibly due to local recommendations, since a systematic school-based fluoride gel programs have been in place since 2004 [17].

### 4.2. European Patients’ Dental Characteristics

Caries prevalence has declined in developed countries due to improved dental healthcare organization, available fluoride products, enhanced oral hygiene, and increased awareness of caries occurrence. While Western and Northern European countries have experienced a decrease in caries rates, Eastern and Central European nations continue to face caries as a public health challenge [18]. In this patient cohort, 44.2% exhibited active carious lesions, consistent with previous findings, with Portugal showing a notably higher proportion (77.9%) [19,20], likely due to our sample characteristics. This disparity may be attributed to Portugal’s lack of coverage for emergency dental care visits [16], resulting in patients mainly seeking treatment for later dental emergencies.

Consistent with previous research [3,7,21,22,23], active carious lesions and caries risk exhibit an inverse association with socioeconomic status across all countries, except in Portugal. This deviation may be explained by the unique characteristics of the Portuguese dentists in our study, who treated a different patient population or by the low representativeness of the Portuguese sample. Additionally, the Portuguese patient cohort had distinct features, such as a significant proportion with systemic conditions that increase the risk of dental caries, long-term consumption of sweetened medications, and a higher prevalence of pits and fissures sealants compared to other countries.

The association between higher caries risk and lower socioeconomic status underscores the importance of addressing oral health inequalities at the structural level, focusing on broader policy initiatives targeting social determinants of health. While these findings confirm inequalities in three European countries—Bulgaria, Greece, and Poland—the results from Portugal may be influenced by our cohort specificity.

The varying durations of history and follow-up periods precluded direct data comparison. Prospective and retrospective investigations suggest that dentists’ practices did not significantly evolve from three years before patient inclusion to one year after. However, a slightly better overall assessment of oral health appeared more frequently during the follow-up period, although these findings require cautious interpretation.

### 4.3. Caries Risk Assessment in Real-Life Dental Practice

Our main finding might be that, despite the likely integration of MID into dental school curricula, the practical implementation of standardized CRA in real-world dental practice poses substantial challenges [24]. The findings reveal that less than 15% of dentists utilize standardized CRA. In the absence of standardized tools, dentists predominantly rely on oral examinations and medical interviews, while nutritional and fluoride intake assessments are utilized to a lesser extent, and salivary or microbiological tests are exceptionally rare. Dentists with specific training in Caries Risk Management show an increased inclination to incorporate risk assessment into their practice. CRA does not automatically translate into Caries Risk Management, underscoring the need for enhancements in the shift toward more specialized dental practices, refined risk assessments, and the integration of automated decision-making. The challenges encountered in implementing standardized CRA in general dentistry exemplify the real-world issues faced by the dental profession

On one hand, our study highlights the critical importance of regular awareness of CRA for enhancing dental professionals’ adherence to Caries Risk Management recommendations, emphasizing the necessity for continuous adaptation within a systemic perspective of dental health.

On the other hand, in real-life practice, standardized risk factor assessments raise questions. Various models are available not only for caries risk assessment but also for evaluating periodontal disease [11], tooth wear [25], and other conditions. Clinicians can only use these assessment tools systematically by compartmentalizing certain diseases, which may not be ideal practice, both at individual and collective levels. As demonstrated by our study, real-life practitioners adopt highly pragmatic approaches, assessing caries risk simply based on clinical examination and medical interview. While this approach may not be optimal for addressing dental caries, it could be beneficial at the patient level. New theoretical models of dental health emphasize the importance of a biopsychosocial approach to health, involving an active consideration of the patient’s life context [26]. This approach enables the identification of all health determinants in a comprehensive manner and facilitates the development of highly personalized strategies.

### 4.4. Strengths and Limits

This study benefits from an ambispective longitudinal design, allowing for a comprehensive understanding of dental practices over time. The inclusion of both prospective and retrospective data offers valuable insights into the evolution of dental care practices. Additionally, the international nature of the cohort, encompassing four European countries, enables comparative analysis across diverse cultural and socioeconomic contexts. The utilization of an online platform for data collection streamlines the process of patient inclusion and data gathering, potentially enhancing the reliability and quality of the collected data. Furthermore, the study collects a diverse range of data, including demographic information, medical history, and oral hygiene practices, providing a comprehensive overview of dental patient profiles.

This study is mainly descriptive including small samples of dentists and patients in subgroups analyses. These results showed trends and should be confirmed with more data and multivariate modeling to explore deeper associations. One other potential limitation of this study is the possibility of dentist selection bias. As participation is voluntary, dentists who choose to participate may possess different characteristics compared to those who do not, potentially biasing the study sample. Additionally, the findings of this study may not be generalizable to regions outside of the four European countries included, limiting the external validity of the results. Another limitation is the reliance on self-reported data from dentists, which may introduce reporting or recall biases. Finally, this study did not account for the impact of the COVID-19 pandemic on dental practices, as data collection had commenced prior to the pandemic. This omission may limit the relevance of the findings in light of recent global events.

## 5. Conclusions

Throughout this study, we observed significant diversity in caries risk management practices among dental clinicians across four European countries, which may partly explain the underuse of standardized CRA tools and highlight the need for further qualitative exploration of underlying factors (such as a lack of time during appointments, dentists’ doubts about the effectiveness or added value of CRA tools in daily practice, perceived complexity or the lack of practicality of the tools). Notably, specific university training correlated with better adherence to guidelines. However, pragmatic approaches, such as relying on clinical examination and medical interviews, were the most prevalent ways to assess caries risk. These findings might underscore clinicians’ adaptability to the complexities of patients’ lives. In conclusion, while standardized risk assessment models exist, clinicians often tailor their approaches to individual patient needs. Embracing this adaptability is crucial for navigating the challenges of caries prevention and treatment effectively. Moving forward, integrating biopsychosocial models into dental practice can enhance patient-centered care and promote oral health equity.

## Figures and Tables

**Table 1:** Dentists’ characteristics by country.

		All N = 51	Bulgaria n = 19	Greece n = 8	Poland n = 14	Portugal n = 10
Men		19 (37.3%)	5 (26.3%)	5 (62.5%)	6 (42.9%)	3 (30.0%)
Age	*Missing value*	*2*	*0*	*1*	*0*	*1*
	Mean (±SD)	44.0 (±12.0)	42.3 (±13.6)	47.1 (±10.4)	46.9 (±13.4)	40.7 (±5.9)
Years of professional practice	*Missing value*	*3*	*1*	*0*	*1*	*1*
	Mean (±SD)	19.0 (±12.7)	18.4 (±14.1)	22.0 (±9.9)	23.7 (±13.0)	10.9 (±7.8)
Years since graduation	*VM*	*5*	*3*	*1*	*0*	*1*
	Mean (±SD)	19.1 (±13.2)	17.1 (±14.5)	24.6 (±11.6)	23.6 (±13.5)	11.6 (±8.1)
Specialized qualification *	Dental surgery	8 (15.7%)	2 (10.5%)	5 (62.5%)	0	1 (10.0%)
	Pediatric dentist	8 (15.7%)	3 (15.8%)	2 (25.0%)	0	3 (30.0%)
	Other *	35 (68.6%)	14 (73.7%)	1 (12.5%)	14 (100%)	6 (60.0%)
Liberal practice	*Missing value*	*1*	*0*	*1*	*0*	*0*
		47 (94.0%)	17 (89.5%)	7 (100%)	13 (92.9%)	10 (100%)
Specific university training on Caries Risk Management	*Missing value*	*1*	*0*	*1*	*0*	*0*
		24 (48.0%)	5 (26.3%)	2 (28.6%)	11 (78.6%)	6 (60.0%)
Dental continuing education	*Missing value*	*1*	*0*	*1*	*0*	*0*
		47 (94.0%)	17 (89.5%)	7 (100%)	14 (100%)	9 (90.0%)
Dentists’ self-perception regarding Caries Risk Management expertise	*Missing value*	*1*	*0*	*1*	*0*	*0*
	Up to date	36 (72.0%)	13 (68.4%)	6 (85.7%)	10 (71.4%)	7 (70.0%)
	Moderately up to date	14 (28.0%)	6 (31.6%)	1 (14.3%)	4 (28.6%)	3 (30.0%)
Practice area	*Missing value*	*1*	*0*	*1*	*0*	*0*
	Rural	9 (18.0%)	4 (21.1%)	1 (14.3%)	2 (14.3%)	2 (20.0%)
	Peri-urban	10 (20.0%)	2 (10.5%)	1 (14.3%)	1 (7.1%)	6 (60.0%)
	Urban	31 (62.0%)	13 (68.4%)	5 (71.4%)	11 (78.6%)	2 (20.0%)
Children patient (as %)	*Missing value*	*1*	*0*	*1*	*0*	*0*
	Mean (±SD)	31.9 (±31.7)	38.7 (±27.3)	32.9 (±46.4)	12.6 (±11.6)	45.5 (±38.2)
Adult patient (as %)	*Missing value*	*1*	*0*	*1*	*0*	*0*
	Mean (±SD)	68.1 (±31.7)	61.3 (±27.3)	67.1 (±46.4)	87.4 (±11.6)	54.5 (±38.2)

* Other included general odontology (n = 7), conservative stomatology/and endodontics (n = 8), orthodontics (n = 3), resident dentist (n = 3), peri odontology (n = 2), esthetic dentistry (n = 1), preventive dentistry and public health (n = 1), prosthodontist (n = 1), and no specialization (n = 1).

**Table 2:** Caries risk management characteristics by country.

		All N = 51	Bulgaria n = 19	Greece n = 8	Poland n = 14	Portugal n = 10
Reliance on Caries Risk Management recommendations	*Missing value*	*2*	*0*	*1*	*0*	*1*
		31 (63.3%)	12 (63.2%)	4 (57.1%)	10 (71.4%)	5 (55.6%)
Frequency of carrying out caries Risk Assessments	*Missing value*	*2*	*0*	*1*	*0*	*1*
	At every patient’s dental visit	28 (57.1%)	9 (47.4%)	7 (100%)	5 (35.7%)	7 (77.8%)
	Only on the first visit	12 (24.5%)	8 (42.1%)	0	2 (14.3%)	2 (22.2%)
Dentist’s self-reported frequency of Caries Risk Assessments—Follow-up details	*Missing value*	*23*	*10*	*1*	*9*	*3*
	For Children	10 (35.7%)	4 (44.4%)	2 (28.6%)	0	4 (57.1%)
	For elderly patients	8 (28.6%)	4 (44.4%)	3 (42.9%)	1 (20.0%)	0
	Other	10 (35.7%)	1 (11.1%)	2 (28.6%)	4 (80.0%)	3 (42.9%)
Dentist’s self-reported non-conduct of Caries Risk Assessments—Follow-up details	*Missing value*	*42*	*17*	*8*	*7*	*10*
	If there is no carie	7 (77.8%)	2 (100%)	0	5 (71.4%)	0
	Other	2 (22.2%)	0	0	2 (28.6%)	0
Mean frequency of assessing patient’s risk for dental caries	*Missing value*	*2*	*0*	*1*	*0*	*1*
	Each visit	13 (26.5%)	2 (10.5%)	5 (71.4%)	2 (14.3%)	4 (44.4%)
	Once a year	26 (53.1%)	12 (63.2%)	1 (14.3%)	8 (57.1%)	5 (55.6%)
	Twice a year	10 (20.4%)	5 (26.3%)	1 (14.3%)	4 (28.6%)	0
Process of Caries Risk Assessment	*Missing value*	*2*	*0*	*1*	*0*	*1*
	Oral exam	48 (98.0%)	18 (94.7%)	7 (100%)	14 (100%)	9 (100%)
	Medical interview	39 (79.6%)	15 (78.9%)	5 (71.4%)	14 (100%)	5 (55.6%)
	Salivary tests	4 (8.2%)	0	0	1 (7.1%)	3 (33.3%)
	CRA (guideline)	4 (8.2%)	2 (10.5%)	0	0	2 (22.2%)
	CRA (software program)	3 (6.1%)	2 (10.5%)	0	1 (7.1%)	0
	Radiographic examinations	33 (67.3%)	7 (36.8%)	7 (100%)	12 (85.7%)	7 (77.8%)
	Nutritional assessment	35 (71.4%)	14 (73.7%)	5 (71.4%)	9 (64.3%)	7 (77.8%)
	Fluoridation	25 (51.0%)	4 (21.1%)	4 (57.1%)	12 (85.7%)	5 (55.6%)
	Fluor intake	20 (40.8%)	4 (21.1%)	4 (57.1%)	10 (71.4%)	2 (22.2%)
	Others	6 (12.2%)	2 (10.5%)	0	2 (14.3%)	2 (22.2%)

**Table 3:** Patients’ characteristics by country.

		All N = 1008	Bulgaria n = 366	Greece n = 283	Poland n = 273	Portugal n = 86
**Men**		443 (43.9%)	140 (38.3%)	129 (45.6%)	130 (47.6%)	44 (51.2%)
Age	*Missing value*	*3*	*2*	*0*	*1*	*0*
	Mean (±SD)	35.2 (±19.7)	35.3 (±18.3)	31.7 (±20.9)	42.4 (±17.7)	24.4 (±19.6)
	[Range]	[0–89]	[0–85]	[0–80]	[6–89]	[7–76]
Socioeconomic status	Low n (%)	48 (4.8)	11 (3.0)	17 (6.0)	6 (2.2)	14 (16.3)
	Medium n (%)	593 (58.8)	213 (58.2)	189 (66.8)	146 (53.5)	45 (52.3)
	High n (%)	227 (22.5)	73 (19.9)	41 (14.5)	113 (41.4)	0
	Unknown n (%)	140 (13.9)	69 (18.9)	36 (12.7)	8 (2.9)	27 (31.4)
Daily teeth brushing by patients n (%)		928 (92.1)	345 (94.3)	234 (82.7)	269 (98.5)	80 (93.0)
Use of fluoride toothpaste by patients n (%)		834 (82.7)	233 (63.7)	249 (88.0)	269 (98.5)	83 (96.5)
Implementation of additional home measures (protective factors: antimicrobial, fluoride mouthrinse, xylitol, calcium, phosphate, etc.) by patients n (%)		474 (47.0)	194 (53.0)	112 (39.6)	136 (49.8)	32 (37.2)
Presence of white spot lesions or enamel defects n (%)		234 (23.2)	78 (21.3)	59 (20.8)	68 (24.9)	29 (33.7)
Presence of deep pits and fissures without sealants n (%)		387 (38.4)	132 (36.1)	124 (43.8)	85 (31.1)	46 (53.5)
Visibility of plaque on teeth n (%)		537 (53.3)	156 (42.6)	167 (59.0)	156 (57.1)	58 (67.4)
Presence of signs of dry mouth n (%)		69 (6.8)	20 (5.5)	16 (5.7)	28 (10.3)	5 (5.8)
Presence of active caries lesions n (%)		446 (44.2)	157 (42.9)	108 (38.2)	114 (41.8)	67 (77.9)
Use of intraoral appliances (orthodontic appliance/prosthesis) by patients n (%)		218 (21.6)	46 (12.6)	74 (26.1)	76 (27.8)	22 (25.6)
Overall subjective assessment of oral dental health by dentists n (%)	High	287 (28.5)	102 (27.8)	44 (15.5)	129 (47.2)	12 (14.0)
	Medium	562 (55.7)	225 (61.5)	168 (59.4)	125 (45.8)	44 (51.1)
	Low	159 (15.8)	39 (10.7)	71 (25.1)	19 (7.0)	30 (34.9)
Assessment of dental caries risk by dentists n (%)	High risk	464 (46.0)	157 (42.9)	116 (41.0)	122 (44.7)	69 (80.2)
	Low risk	544 (54.0)	209 (57.1)	167 (59.0)	151 (55.3)	17 (19.8)
Provision of counseling (oral hygiene instruction, etc.) by dentists n (%)		891 (88.4)	362 (98.9)	261 (92.2)	213 (78.0)	55 (64.0)
Assessment of patient’s attitude towards care and willingness to cooperate by dentists n (%)	High	566 (56.2)	250 (68.3)	130 (45.9)	161 (59.0)	25 (29.1)
	Medium	347 (34.4)	100 (27.3)	125 (44.2)	94 (34.4)	28 (32.5)
	Low	65 (6.4)	16 (4.4)	25 (8.8)	18 (6.6)	6 (7.0)
	Unknown	30 (3.0)	0	3 (1.1)	0	27 (31.4)
Recommended recall interval for next oral health review by dentists n (%)		921 (91.4)	365 (99.7)	272 (96.1)	199 (72.9)	85 (98.8)

**Table 4:** Last 3 years of history of patients.

Last 3 Years		All N = 627	Bulgaria n = 138	Greece n = 165	Poland n = 268	Portugal n = 56
Incidence of active caries during the period n (%)		415 (66.3)	78 (56.5)	107 (64.8)	179 (67.0)	51 (91.1)
Overall assessment of oral dental health n (%)	High	174 (27.8)	40 (29.0)	14 (8.5)	114 (42.5)	6 (10.7%)
	Medium	346 (55.2)	82 (59.4)	103 (62.4)	133 (49.6)	28 (50.0%)
	Low	107 (17.1)	16 (11.6)	48 (29.1)	21 (7.8)	22 (39.3%)
Reassessment of dental caries risk n (%)		342 (54.5)	95 (68.8)	41 (24.8)	155 (57.8)	51 (91.1)
Caries care provided during the last 3 years						
Fluoride application n (%)		326 (52.0)	4 (2.9)	89 (53.9)	181 (67.5)	52 (92.9)
Restorations n (%)		470 (75.0)	109 (79.0)	102 (61.8)	208 (77.6)	51 (91.1)
Pits and fissures sealants n (%)		134 (21.4)	14 (10.1)	55 (33.3)	15 (5.6)	50 (89.3)
Sealant control and repair n (%)		49 (7.8)	11 (8.0)	9 (5.5)	14 (5.2)	15 (26.8)
Remineralization n (%)		81 (12.9)	6 (4.3)	2 (1.2)	54 (20.1)	19 (33.9)
Infiltration n (%)		5 (0.8)	4 (2.9)	0	1 (0.4)	0
Other n (%)		219 (34.9)	52 (37.7)	25 (15.2)	105 (39.2)	37 (66.1)
**Patient education during the last 3 years**						
Provision of counseling (oral hygiene instruction, etc.) by dentists n (%)		496 (79.1)	131 (94.9)	140 (84.8)	177 (66.0)	48 (85.7)
Assessment of patient’s attitude towards care and willingness to cooperate by dentists n (%)	High	241 (38.5)	73 (52.9)	33 (20.0)	125 (46.8)	10 (17.9%)
	Medium	256 (40.9)	58 (42.0)	82 (49.7)	103 (38.6)	13 (23.2%)
	Low	65 (10.4)	4 (2.9)	34 (20.6)	17 (6.4)	10 (17.9%)
Recommended recall interval for next oral health review by dentists n (%)		504 (80.5)	134 (97.1)	136 (82.4)	179 (67.0)	55 (98.2)

**Table 5:** Twelve months of patient follow-up.

12 Months Follow-Up		All N = 731	Bulgaria n = 281	Greece n = 236	Poland n = 214
Incidence of active caries during the period n (%)		196 (26.8)	85 (30.2)	48 (20.3)	63 (29.4)
Overall assessment of oral dental health n (%)	High	237 (32.4)	97 (34.5)	43 (18.2)	97 (45.3%)
	Medium	341 (46.7)	156 (55.5)	93 (39.4)	92 (43.0%)
	Low	153 (20.9)	28 (10.0)	100 (42.4)	25 (11.7%)
Reassessment of dental caries risk n (%)		412 (56.4)	176 (62.6)	117 (49.6)	119 (55.6)
Caries care provided during the 12 months follow-up					
Fluoride application n (%)		226 (30.9)	14 (5.0)	110 (46.6)	102 (47.7)
Restorations n (%)		279 (38.2)	126 (44.8)	71 (30.1)	82 (38.3)
Pits and fissures sealants n (%)		35 (4.8)	11 (3.9)	21 (8.9)	3 (1.4)
Sealant control and repair n (%)		30 (4.1)	10 (3.6)	12 (5.1)	8 (3.7)
Remineralization n (%)		102 (14.0)	19 (6.8)	37 (15.7)	46 (21.5)
Infiltration n (%)		1 (0.1)	0	0	1 (0.5)
Other n (%)		172 (23.5)	63 (22.4)	42 (17.8)	67 (31.3)
Patient education during the 12 months follow-up					
Provision of counseling (oral hygiene instruction, etc.) by dentists n (%)		524 (71.7)	239 (85.1)	162 (68.6)	123 (57.5)
Assessment of patient’s attitude towards care and willingness to cooperate by dentists n (%)	High	272 (37.2)	128 (45.6)	64 (27.1)	80 (37.4%)
	Medium	282 (38.6)	119 (42.3)	84 (35.6)	79 (36.9%)
	Low	42 (5.7)	11 (3.9)	20 (8.5)	11 (5.1%)
Recommended recall interval for next oral health review by dentists n (%)		512 (70.0)	252 (89.7)	150 (63.6)	110 (51.4)

## Data Availability

The data that support the findings of this study are available from the corresponding author upon reasonable request.

## Supplementary Materials

The following supporting information can be downloaded at: https://www.mdpi.com/article/10.3390/dj14020126/s1, Supplementary Table S1: Preventive measures characteristics, description by country. Supplementary Table S2: Patients’ characteristics with carious risk impact, description by country.
