# Supplementary material for: Caries Risk Assessment and Management in Europe: The Multi-Country Observational CARMEN Study
Source: Dent J (Basel). 2026 Feb 23;14(2):126. doi: 10.3390/dj14020126 (PMC12939442; doi:10.3390/dj14020126)
Supplement: Supplementary file 1 [file dentistry-14-00126-s001.zip › dentistry-3602869-supplementary.pdf]

Supplementary Table S1: Preventive measures characteristics, description by country

		All<br>N=51	Bulgaria<br>N=19	Greece<br>N=8	Poland<br>N=14	Portugal<br>N=10
Fluor systemic prescription	*Missing value*	*2*	*0*	*1*	*0*	*1*
		0	0	0	0	0
Topical applications	*Missing value*	*2*	*0*	*1*	*0*	*1*
		38 (77.6%)	10 (52.6%)	5 (71.4%)	14 (100%)	9 (100%)
Topical applications: for patients with	*Missing value*	*0*	*0*	*0*	*0*	*0*
	High risk caries	15 (39.5%)	6 (60.0%)	1 (20.0%)	3 (21.4%)	5 (55.6%)
	Both	23 (60.5%)	4 (40.0%)	4 (80.0%)	11 (78.6%)	4 (44.4%)
Type of sealants materials	*Missing value*	*2*	*0*	*1*	*0*	*1*
	Toothpaste	42 (85.7%)	15 (78.9%)	7 (100%)	13 (92.9%)	7 (77.8%)
	Varnish	35 (71.4%)	8 (42.1%)	5 (71.4%)	14 (100%)	8 (88.9%)
	Pit and fissure sealants	42 (85.7%)	18 (94.7%)	4 (57.1%)	11 (78.6%)	9 (100%)
	Dental gutter	13 (26.5%)	1 (5.3%)	4 (57.1%)	4 (28.6%)	4 (44.4%)
Provide diet counseling	*Missing value*	*2*	*0*	*1*	*0*	*1*
		41 (83.7%)	14 (73.7%)	5 (71.4%)	14 (100%)	8 (88.9%)
Provide oral hygiene education	*Missing value*	*2*	*0*	*1*	*0*	*1*
		47 (95.9%)	17 (89.5%)	7 (100%)	14 (100%)	9 (100%)
Oral hygiene	*Missing value*	*2*	*0*	*1*	*0*	*1*
	Toothpaste	49 (100%)	19 (100%)	7 (100%)	14 (100%)	9 (100%)
	Toothbrush	47 (95.9%)	19 (100%)	7 (100%)	13 (92.9%)	8 (88.9%)
	Mouthwash	45 (91.8%)	19 (100%)	7 (100%)	13 (92.9%)	6 (66.7%)
	Interdental brush	44 (89.8%)	16 (84.2%)	7 (100%)	14 (100%)	7 (77.8%)
	Dental floss	47 (95.9%)	17 (89.5%)	7 (100%)	14 (100%)	9 (100%)
Record diet chart	*Missing value*	*2*	*0*	*1*	*0*	*1*
		7 (14.3%)	2 (10.5%)	2 (28.6%)	0	3 (33.3%)
Diet diary	*Missing value*	*2*	*0*	*1*	*0*	*1*
		9 (18.4%)	2 (10.5%)	5 (71.4%)	1 (7.1%)	1 (11.1%)
Practices adapted according to a caries risk assessment	*Missing value*	*2*	*0*	*1*	*0*	*1*
		42 (85.7%)	16 (84.2%)	6 (85.7%)	11 (78.6%)	9 (100%)

Supplementary Table S2: Patients' characteristics with carious risk impact, description by country

	**All**<br>**N=1008**	**Bulgaria**<br>**N=366**	**Greece**<br>**N=283**	**Poland**<br>**N=273**	**Portugal**<br>**N=86**
Systemic conditions that could increase the patient's risk of developing dental caries (such as Amelogenesis imperfecta,)	67 (6.6%)	11 (3.0%)	22 (7.8%)	12 (4.4%)	22 (25.6%)
Physical conditions that may reduce the patient's ability to maintain their oral health, or that may complicate dental treatment (e.g. disability)	19 (1.9%)	1 (0.3%)	6 (2.1%)	9 (3.3%)	3 (3.5%)
Long-term consumption of sweetened medication	64 (6.3%)	17 (4.6%)	9 (3.2%)	20 (7.3%)	18 (20.9%)
Recent and previous caries experience	804 (79.8%)	252 (68.9%)	212 (74.9%)	262 (96.0%)	78 (90.7%)
High caries in mother and siblings	608 (60.3%)	253 (69.1%)	103 (36.4%)	207 (75.8%)	45 (52.3%)
Pits and fissures sealants	186 (18.5%)	38 (10.4%)	62 (21.9%)	32 (11.7%)	54 (62.8%)
High and/or frequent sugar intake between meals (sweet snacks, drinks, sweets,...)	398 (39.5%)	147 (40.2%)	107 (37.8%)	106 (38.8%)	38 (44.2%)
Daily tobacco use	178 (17.7%)	75 (20.5%)	57 (20.1%)	38 (13.9%)	8 (9.3%)
Daily alcohol use	57 (5.7%)	35 (9.6%)	11 (3.9%)	6 (2.2%)	5 (5.8%)
Caries care provided during the inclusion visit: Fluoride application	259 (25.7%)	12 (3.3%)	105 (37.1%)	100 (36.6%)	42 (48.8%)
Caries care provided during the inclusion visit: Restorations	535 (53.1%)	237 (64.8%)	140 (49.5%)	133 (48.7%)	25 (29.1%)
Caries care provided during the inclusion visit : Pits and fissures sealants	85 (8.4%)	32 (8.7%)	33 (11.7%)	5 (1.8%)	15 (17.4%)
Caries care provided during the inclusion visit : Sealant control and repair	45 (4.5%)	26 (7.1%)	7 (2.5%)	8 (2.9%)	4 (4.7%)
Caries care provided during the inclusion visit: Remineralisation	86 (8.5%)	14 (3.8%)	34 (12.0%)	34 (12.5%)	4 (4.7%)
Caries care provided during the inclusion visit: Infiltration	15 (1.5%)	9 (2.5%)	5 (1.8%)	1 (0.4%)	0
Caries care provided during the inclusion visit: Other	291 (28.9%)	99 (27.0%)	79 (27.9%)	89 (32.6%)	24 (27.9%)
